# Modulation of lignin biosynthesis for drought tolerance in plants

**DOI:** 10.3389/fpls.2023.1116426

**Published:** 2023-04-20

**Authors:** Su Jeong Choi, Zion Lee, Sohyun Kim, Eui Jeong, Jae Sung Shim

**Affiliations:** School of Biological Sciences and Technology, Chonnam National University, Gwangju, Republic of Korea

**Keywords:** plants, phenylpropanoid, lignin, transcriptional regulation, drought tolerance

## Abstract

Lignin is a complex polymer that is embedded in plant cell walls to provide physical support and water protection. For these reasons, the production of lignin is closely linked with plant adaptation to terrestrial regions. In response to developmental cues and external environmental conditions, plants use an elaborate regulatory network to determine the timing and location of lignin biosynthesis. In this review, we summarize the canonical lignin biosynthetic pathway and transcriptional regulatory network of lignin biosynthesis, consisting of NAC and MYB transcription factors, to explain how plants regulate lignin deposition under drought stress. Moreover, we discuss how the transcriptional network can be applied to the development of drought tolerant plants.

## Introduction

As sessile organisms, plants encounter rapid or incremental changes in their surrounding environmental conditions, collectively called environmental stresses, during their growth. Environmental stresses affect various parts of plant development, growth, and reproduction (Nakashima et al., 2014; Takahashi et al., 2020), and extreme stresses trigger severe damage during vegetative and reproductive growth. Therefore, plants must recognize and respond to their changing environment to ensure their survival. For that reason, plants have evolved to utilize multiple signaling networks that mediate structural, chemical, and molecular protection against environmental stresses. Among these environmental stresses, drought represents a major challenge for crop production in the context of current climate changes and regional water shortages (Tardieu et al., 2018). Drought significantly reduces regional water availability, leading to competition between agricultural, industrial, and residential water usage. In addition, current climate changes exacerbate droughts by increasing their frequency, duration, and severity. Therefore, it is necessary to develop strategies to increase or maintain agricultural crop production with reduced water availability by adapting cultivation systems and generating new plant varieties with enhanced drought tolerance (Tester and Langridge, 2010; Tardieu et al., 2018). To achieve this goal, we need to precisely understand the drought-induced molecular changes that trigger drought resistance in plants.

To overcome drought stress, plants have developed three drought resistance mechanisms: drought avoidance, drought tolerance, and drought escape (Lawlor, 2013; Carraro and Di Iorio, 2022). Drought avoidance, which mainly occurs under mild or moderate drought conditions, involves various responses related to maintaining water levels for basal growth; these responses include stomatal closure, leaf rolling, the accumulation of water-preservable metabolites, increases in root growth, and the enhancement of water storage capacity. On the other hand, drought tolerance describes an ability of plants to maintain a certain level of physiological activity under severe drought conditions by reducing drought-induced oxidative and osmotic damages. Finally, drought escape is a strategy in which plants complete their life cycle before the onset of drought stress. Based on physiological output, drought-induced xylem differentiation and increases in lignin content can be categorized as either drought avoidance or tolerance mechanisms. Plants can also use more than one of these mechanisms to overcome drought stress depending on their developmental stage and the drought severity.

Lignin is a complex and heterogeneous polymer consisting of phenylpropanoid subunits, and it is a component of specialized cell walls, which become rigid and water-impermeable with the addition of lignin (Fraser and Chapple, 2011; Xie et al., 2018). Since lignin deposition requires a high and irreversible input of carbon sources, lignin deposition is tightly regulated during plant development through transcriptional, posttranscriptional, and posttranslational processes (Gou et al., 2018; Rao and Dixon, 2018; Cesarino, 2019). In addition to playing a role in plant development, lignin is also involved in plant responses to various biotic and abiotic stresses (Barros et al., 2015; Cesarino, 2019). Although the connection between lignin and drought has been demonstrated in various plants, several studies report changes in lignin content and composition under drought conditions without investigating the underlying molecular regulatory processes. However, recent studies have provided multiple lines of evidence suggesting that enhanced lignin deposition through either transcriptional or enzymatic modifications increases drought tolerance in plants (Lee et al., 2016; Bang et al., 2019; Cesarino, 2019). It has also been proposed that master transcriptional regulators of lignin biosynthesis are activated by drought and ABA *via* transcriptional regulation or posttranslational modification (Liu et al., 2021a; Bang et al., 2022; Jung et al., 2022). Increases in lignin content in response to drought could result from lignin deposition in new xylem or existing vascular cells (Srivastava et al., 2015; Liu et al., 2021b; Ramachandran et al., 2021). These findings propose molecular mechanisms involved in drought-mediated changes in xylem differentiation and corresponding lignin deposition. In addition, they suggest that enhanced lignin accumulation that increases physical defenses against drought can be leveraged as another strategy to develop drought tolerant plants. This review aims to introduce the transcriptional regulation of lignin biosynthesis that occurs during secondary cell wall development and drought and summarize recent achievements regarding the development of drought tolerant plants by transcriptional or enzymatic modulation of lignin biosynthesis. Finally, we discuss important aspects that should be considered when developing drought tolerant plants through enhanced lignin deposition.

## The importance of lignin in plants

Lignin is a cell wall–binding phenolic polymer that is synthesized by almost all plants (Weng and Chapple, 2010; Renault et al., 2017). Lignin is mainly located in the secondary cell wall, where it provides the physical strength required for supporting plant structures. In addition, lignin is essential in supporting the transport of water and minerals through xylem. Due to these physical functions, lignin, together with other phenylpropanoid metabolites, is regarded as an important metabolite in the evolution of land plants (Weng and Chapple, 2010). Early land plants first acquired an ability to produce phenylpropanoid metabolites to protect themselves from UV irradiation—a formidable challenge hindering successful migration to land (Lowry et al., 1980). However, until the rise of tracheophytes, early land plants could not synthesize lignin. Uniquely, tracheophytes could deposit lignin in their cell wall to increase mechanical support and strengthen water-conducting cells for long-distance water transport, both of which were necessary for their growth in size (Weng and Chapple, 2010). Thus, the acquisition of the phenylpropanoid pathway and production of lignin were essential in enabling the dominance of tracheophytes in this terrestrial environment.

Due to their importance for water transport, large amounts of lignin are deposited in xylem vascular tissues such as protoxylem, metaxylem, and fibers (Barros et al., 2015). Unlike lignification in protoxylem and metaxylem, lignification of fibers is not associated with water transport. Lignin is also deposited in the endodermal cells that form a Casparian strip (Naseer et al., 2012). Lignin deposition in xylem vascular tissues and Casparian strip is important for water uptake from soil and transport to shoots. Lignins are mainly composed of three hydroxycinnamyl alcohol monomers—*p*-coumaryl, coniferyl, and sinapyl alcohols—which produce *p*-hydroxylphenyl (H), guaiacyl (G), and syringyl (S) monolignol units, respectively (Vanholme et al., 2010). Lignin composition varies between cell types and cell wall layers. For example, tracheary elements (TEs) and Casparian strips are predominantly enriched with G units, while fibers are enriched with S-units (Fukushima and Terashima, 1991; Naseer et al., 2012; Blaschek et al., 2020; Ménard et al., 2022). In addition, the proportion of monomers with different aliphatic residues such as alcohol (X_CHOH_) or aldehyde (X_CHO_) also differs between cell types. For one, the proportion of aldehyde residues (X_CHO_) is higher in metaxylem than protoxylem, and X_CHO_ is higher in primary cell walls and middle lamellae compared to secondary cell walls (Hänninen et al., 2011; Blaschek et al., 2020). In addition to being regulated by cell type, the incorporation of lignin monomers varies temporally in plants. H and G monomers are incorporated in the early maturation stage, while S and X_CHO_ are deposited in the later maturation stage (Blaschek et al., 2020; Ménard et al., 2022). Overall, lignin deposition in xylem vascular tissues is beneficial for plant survival under drought conditions (Ménard et al., 2022). Lignin deposition is also known to facilitate plant protection against pathogen invasion. In response to pathogen attack and wounding damage, lignin with a higher proportion of H units is deposited in cell walls at the site of infection or damage (Malinovsky et al., 2014; Reyt et al., 2021), helping plants minimize dehydration and strengthen physical barriers to prohibit pathogen entrance into cells (Bhuiyan et al., 2009). These differences in lignin composition between different cell types suggest that lignin polymerization is tightly regulated so that plants can adjust the flexibility of vascular tissues according to developmental requirements and environmental conditions.

## Lignin biosynthesis

**Figure 1:**
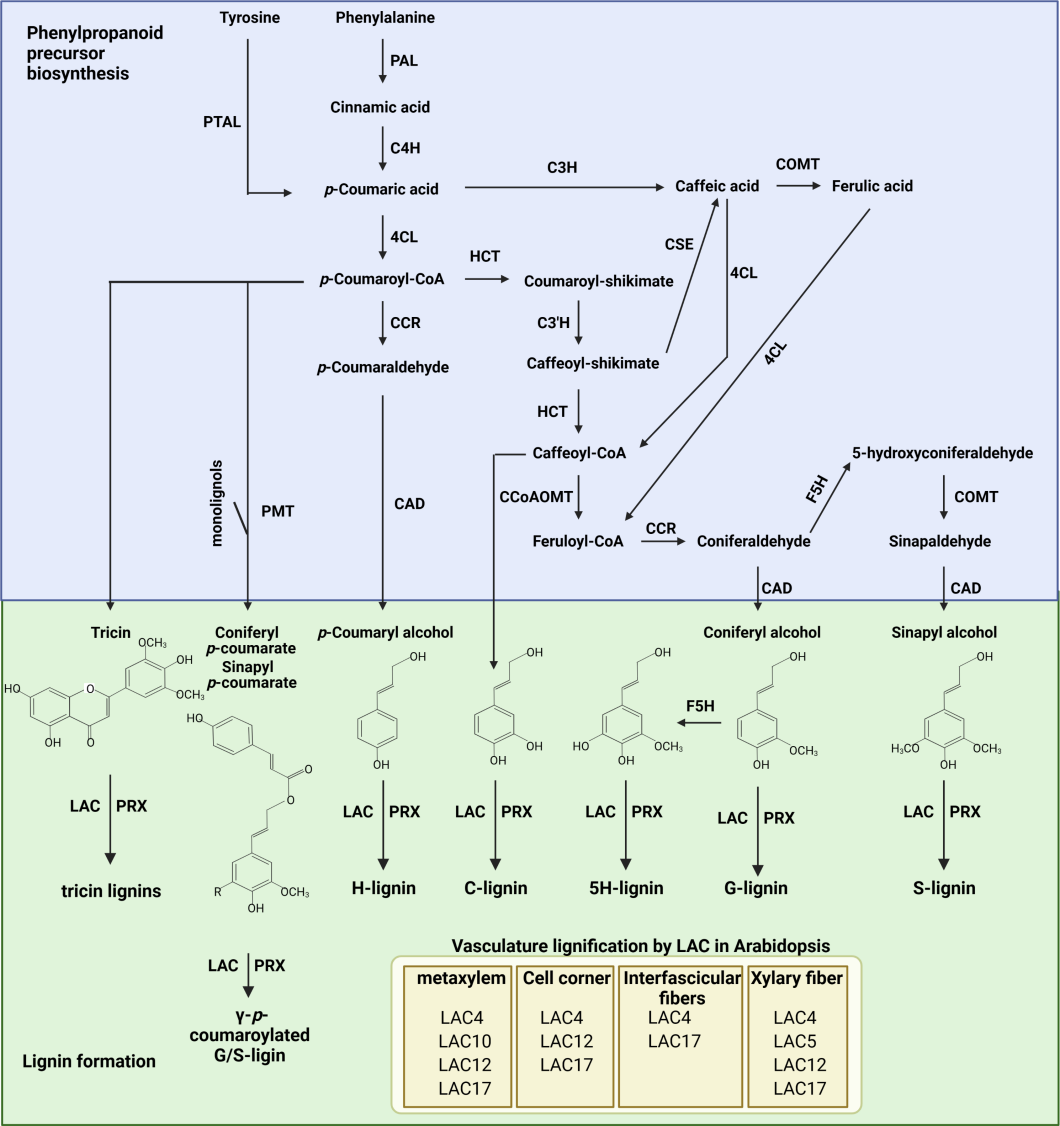
The lignin biosynthesis pathway from phenylalanine in plants. PAL, phenylalanine ammonia-lyase; PTAL, phenylalanine tyrosine ammonia lyase; C4H, cinnamic acid 4-hydroxylase; 4CL, 4-Coumarate: CoA ligase; CCR, cinnamoyl-CoA reductase; HCT, quinate/shikimate *p*-hydroxycinnamoyltransferase; C3’H, *p*-coumaroylshikimate 3’-hydroxylase; C3H, coumarate 3-hydroxylase; CSE, caffeoyl shikimate esterase; CCoAOMT, caffeoyl-CoA *O*-methyltransferase; F5H, ferulate 5-hydroxylase; COMT, caffeic acid *O*-methyltransferase; CAD, cinnamyl alcohol dehydrogenase; PMT, *p*-coumaroyl-CoA:monolignol transferase; LAC, laccase; PRX, peroxidase. Arabidopsis LACs involved in lignification of vascular tissue are illustrated on the bottom. The figure was created with Biorender (http://biorender.com).

Lignin biosynthesis can be divided into three major steps: monolignol biosynthesis through the phenylpropanoid biosynthesis pathway in the cytoplasm, transport of lignin precursors into apoplasts, and its polymerization in the cell wall. Monolignols are synthesized through the phenylpropanoid pathway (Vanholme et al., 2010; Fraser and Chapple, 2011; Xie et al., 2018), which is required for the biosynthesis of multiple phenolic compounds in addition to lignin (Fraser and Chapple, 2011; Zhang et al., 2015; Zhang et al., 2021). Monolignol biosynthesis begins with the deamination of phenylalanine through phenylalanine ammonia-lyase (PAL), producing cinnamic acid. Due to its critical role in phenylpropanoid biosynthesis, function of PAL has been extensively studied in various plants (Wanner et al., 1995; Zhu et al., 1995; Chang et al., 2008; Zhan et al., 2022). In Arabidopsis (*Arabidopsis thaliana*), mutation of *PAL1* and *PAL2* led to a significant decrease in phenylpropanoids and lignin (Rohde et al., 2004). Cinnamate 4-hydroxylase (C4H), a P450 monooxygenase, introduces a hydroxyl group into the phenyl ring of cinnamic acid, producing *p*-coumaric acid. Mutation of *C4H* altered various aspects of metabolic flow, decreasing the abundance of several end-products of the phenylpropanoid pathway (Schilmiller et al., 2009). In addition, grasses can generate *p*-coumaric acid from tyrosine through the phenylalanine tyrosine ammonia lyase (PTAL) enzyme (Barros et al., 2016). *p*-Coumaric acid can be further hydroxylated at the C3 position by coumarate 3-hydroxylase (C3H) to generate caffeic acid (Barros et al., 2019), which can also be generated by caffeoyl shikimate esterase (CSE) from caffeoyl-shikimate (Vanholme et al., 2013). The hydroxyl group at the C3 position of caffeic acid is then methylated by cinnamyl alcohol dehydrogenase (COMT), producing ferulic acid. Subsequently, a carboxyl group of *p*-coumaric acid and ferulic acid is reduced into alcohol by 4-coumarate: CoA ligase (4CL), cinnamoyl-CoA reductase (CCR), and cinnamyl alcohol dehydrogenase (CAD). *p*-Coumaroyl-CoA is also converted into caffeoyl-CoA by quinate/shikimate *p*-hydroxylcinnamoyltransferase (HCT) and *p*-coumaroylshikimate 3’-hydroxylase (C3’H). Through caffeoyl-CoA *O*-methyltransferase (CCoAOMT), caffeoyl-CoA is further converted to feruloyl-CoA, which is then reduced by CCR to form coniferaldehyde. Coniferaldehyde is hydroxylated by ferulate 5 hydroxylase (F5H), producing 5-hydroxylconiferaldehyde, and then converted into sinapyl alcohol by caffeic acid *O*-methyltransferase (COMT) and cinnamyl alcohol dehydrogenase (CAD). Through these sequential enzymatic reactions, cinnamic acid is converted into three phenolic precursors (Fraser and Chapple, 2011; Xie et al., 2018) (**Figure 1**). In addition to these three general precursors of lignin, C, 5H, tricin, and coumaroylated G/S monomers are also synthesized in plants (Petrik et al., 2014; Zhao, 2016; Lam et al., 2021).

The phenolic precursors synthesized in the cytoplasm are exported into apoplasts, where they are polymerized to form lignin through a radical reaction catalyzed by lignin formation enzymes, specifically peroxidase (PRX) and laccases (LACs), in the cell wall (Berthet et al., 2011; Shigeto et al., 2015; Blaschek and Pesquet, 2021) (**Figure 1**). Since lignin accumulation in xylem cells occurs after committed programed cell death (post-mortem lignification), cell type–specific lignification can be achieved by the specific localization of lignin formation enzymes. Although various PRXs and LACs are both heavily involved in lignin deposition in plants, their spatial distributions can differ. For example, AtLAC4, AtLAC17, and AtPRX72 were found to be localized to the thick secondary cell wall of xylem, whereas AtLAC4, AtPRX64, and AtPRX71 were detected in fiber cells (Hoffmann et al., 2020). Shigeto et al. (2015) found that *AtPRX2*, *AtPRX25*, and *AtPRX71* mediate the lignification of Arabidopsis stems. Mutation of these genes reduces lignin content up to 25%. In addition, it has been reported that *AtPRX17* is required for the lignification of leaves, stems, flowers, and siliques in Arabidopsis plants (Cosio et al., 2017), while lignification of Casparian strips is largely mediated by *AtPRX64* (Lee et al., 2013). On the other hand, AtPRX72 is involved in lignification of both vascular tissues and stems, with mutation of *AtPRX72* causing the collapse of some xylem cells and reduced inflorescence stem height (Herrero et al., 2013). Overall, PRXs seem to display different affinity toward particular monolignols. For example, AtPRX2 and AtPRX71 prefer the S unit over the G unit for oxidation (Shigeto et al., 2014). Similarly, genetic analysis using multiple *LAC* mutants brought further support to the idea that lignin deposition is mediated by cell type–specific LACs (Blaschek et al., 2023). Mutation of *LAC4*, *LAC11*, and *LAC17*, which are highly expressed in lignifying tissue, significantly reduced lignin accumulation in stems and roots (Zhao et al., 2013). More specifically, *LAC4* plays a prominent role in lignin deposition in metaxylem, while *LAC17* is important for lignin deposition in interfascicular and xylary fibers. In addition to these *LAC* genes, *LAC5*, *LAC10*, and *LAC12* nonredundantly alter lignin accumulation in different lignified cell types (Blaschek et al., 2023). It is also worth mentioning that *LAC4*, *LAC5*, *LAC10*, *LAC12*, and *LAC17* are less important for lignin accumulation in protoxylem than other lignified cell types. Similarly, mutation of the above five *LAC*s did not affect mechanical strengthening of secondary xylem TEs (Blaschek et al., 2023). These results indicate that additional *LAC*s are involved in lignin accumulation in protoxylem and secondary xylem. In addition to lignin accumulation, *LAC* affects cell type specific ring structure and terminal residue composition in lignin (Blaschek et al., 2023). *LAC4* and *LAC17* predominantly regulate accumulation of S residue in interfascicular fibers, while *LAC4*, *LAC12*, and *LAC17* are involved in accumulation of S residue in xylary fibers. In case of protoxylem and metaxlyem, *LAC4*, *LAC5*, *LAC10*, *LAC12*, and *LAC17* show functional redundancy for regulation of S/G ratio. Proportion of terminal residues [the levels of G subunits with aldehyde (G_CHO_) versus alcohol residues (G_CHOH_)] is also regulated by different set of *LAC*s depending on cell types (Blaschek et al., 2023). *LAC4* and *LAC17* is important for incorporating G_CHO_ in interfascicular fibers, while *LAC4, LAC5, LAC12*, and *LAC17* contribute to G_CHO_ accumulation in xylary fibers. Different with these cell types, G_CHO_/G_CHOH_ ratio in protoxylem and metaxylem is not affected by *LAC4*, *LAC5*, *LAC10*, *LAC12*, and *LAC17.* These unique spatial distributions and nonredundant contributions suggest that different sets of PRXs and LACs mediate lignification depending on the tissue or cell type. Interestingly, AtLAC19, AtPRX42, AtPRX52, and AtPRX71 have been detected in non-lignified tissues. This result indicates that another factor, specifically apoplastic accumulation of hydrogen peroxide either by NADPH oxidase or RBOH, might be required for lignin deposition in addition to PRX and LAC activity (Hoffmann et al., 2020).

In leveraging these biosynthetic pathways, many efforts have been made to control the chemical composition of lignin in plants. For example, suppression of *HCT* and *C3’H* in alfalfa has been attempted in efforts to enrich lignin with H units, which helps to reduce biomass recalcitrance for biological conversion processes (Shadle et al., 2007; Pu et al., 2009). In switchgrass, suppression of both *F5H* and *COMT* significantly reduced lignin S units but increased G units (Wu et al., 2019). To reduce the proportion of G units, the *CCoAOMT* gene can be targeted for suppression. For example, down-regulation of *CCoAOMT* in *Pinus radiate* produced up to a 10-fold increase in the H/G ratio (Wagner et al., 2011). Similarly, RNAi-mediated suppression of *CCoAOMT* in maize reduced the ratio of G units in lignin (Li et al., 2013). In poplar, overexpression of *F5H* driven by a *C4H* promoter increased S units in lignin by 97.5% (Stewart et al., 2009). These studies evidence how the physical and chemical properties of lignin can be changed through regulation of phenylpropanoid biosynthetic genes.

## Transcriptional regulation of lignin biosynthesis in plants

The regulation of lignin deposition is crucial for successful development in plants. Accumulation of lignin in a given cell is a consequence of metabolic flux from carbon sources through the shikimate, phenylpropanoid, and monolignol biosynthesis pathways. The rate of flux through the pathway is affected by the relative amount or activity of key regulatory enzymes. For that reason, many efforts have been made to elucidate the transcriptional regulation of phenylpropanoid and lignin formation enzymes. Studies examining the regulation of *PAL* transcription have provided several important insights into the transcriptional regulation of phenylpropanoid biosynthesis in plants (Hatton et al., 1995; Kim et al., 2014; Xie et al., 2018). Since PAL activity is required for synthesis of various phenylpropanoid compounds, which are important for plant development and responses to environmental conditions, expression of *PAL* genes has to be regulated by various internal and external signals. During plant development, the PAL promoter is activated in differentiating xylem cells, cortices, root tips, and pollen (Bevan et al., 1989; Hatton et al., 1995). Promoter deletion analysis has found that the minimum sequence required for tissue-specific expression of *PAL* is localized within -254 bp from the transcriptional start site. The specific motifs found from the *PAL* promoter are known as AC elements due to the enrichment of adenosine and cytosine (Hatton et al., 1995). These AC elements have also been identified from the promoters of other phenylpropanoid and lignin biosynthesis genes. These observations suggest that expression of phenylpropanoid and lignin biosynthetic genes is co-regulated by specific types of transcription factors. In agreement with the prediction of Hatton et al. (1995), it was found that certain AC elements act as MYB protein binding sites. Among the MYB transcription factors regulating phenylpropanoid and lignin biosynthesis, MYB46 is well-studied in plants (Zhao et al., 2005). MYB46 functions as a master regulator in secondary cell wall formation in Arabidopsis. To regulate the secondary cell wall thickening of xylem cells, *MYB46* is predominantly expressed in fibers and metaxylem within stems. Similarly, MYB83, a close homolog of MYB46, was identified as a key regulator of secondary cell wall biosynthesis (McCarthy et al., 2009). Ectopic expression of *MYB46* under the control of a dexamethasone-inducible promoter revealed that *MYB46* controls expression of multiple *MYB* transcription factors (*MYB43*, *MYB52*, *MYB54*, *MYB58*, and *MYB63*), all of which are activators of secondary cell wall biosynthesis (Ko et al., 2009). In addition, promoter analysis revealed that MYB46 binds to a secondary wall MYB-responsive element (SMRE), which resembles a previously reported AC element. MYB46 activates expression of MYB transcription factors as well as phenylpropanoid biosynthetic genes, including *PAL1*, *C4H*, *4CL1*, *HCT*, *CCoAOMT*, *CCR1, F5H1*, and *CAD6* (Zhong and Ye, 2012; Kim et al., 2014). Poplar *PtrMYB3* and *PtrMYB20*, functional orthologs of Arabidopsis *MYB46* and *MYB83*, also activate expression of genes related to secondary cell wall biosynthesis and deposition of lignin through binding to SMRE elements (McCarthy et al., 2010; Zhong et al., 2013). *MYB58* and *MYB63*, direct targets of MYB46 and MYB83, also activate lignin biosynthetic genes through AC elements (Zhou et al., 2009). Similarly, *MYB20*, *MYB42*, *MYB43*, and *MYB85* regulate phenylalanine and lignin biosynthetic genes during secondary cell wall formation (Geng et al., 2019).

Like MYB transcription factors, NAC transcription factors are involved in regulating lignin biosynthesis. *SECONDARY WALL-ASSOCIATED NAC DOMAIN PROTEIN 1* (*SND1*) is the first NAC transcription factor to have been identified in secondary cell wall biosynthesis of fiber cells, which provide structural support in plants (Zhong et al., 2006). *SND1* is specifically expressed in interfascicular fibers and xylary fibers in the stem. Overexpression or dominant repression of *SND1* significantly altered secondary cell wall thickening of fibers. In particular, overexpression of *SND1* is known to induce ectopic deposition of lignin in the secondary wall of epidermal and mesophyll cells, suggesting that proper developmental regulation of *SND1* is important for fiber development. These observations suggest that *SND1* is a key regulator of secondary cell wall biosynthesis in fibers (Zhong et al., 2006). Consistent with this idea, it was found that *MYB46* and *MYB83* are direct targets of SND1 (Zhong et al., 2007a; McCarthy et al., 2009). However, mutation of *SND1* did not alter secondary cell wall development (Zhong et al., 2007b). Based on this observation, *NAC SECONDARY WALL THICKENING PROMOTING FACTOR1* (*NST1*), a homolog of *SND1*, was identified in Arabidopsis. Double mutation of *SND1* and *NST1* significantly reduced secondary cell wall development and lignin accumulation in stem fiber cells (Zhong et al., 2007b). It has also been reported that additional NAC transcription factors [*NST2*, *NST3*, *VASCULAR-RELATED NAC DOMAIN 6* (*VND6*), and *VND7*] are crucial for secondary cell wall development in fiber cells (NST2 and SND1/NST3), protoxylem (VND7), and metaxylem (VND6) (Mitsuda et al., 2005; Mitsuda et al., 2007; Ohashi-Ito et al., 2010; Yamaguchi et al., 2011). These results illustrate a representative hierarchical transcriptional regulatory network built around secondary cell wall development and lignin biosynthesis that consists of NAC and MYB transcription factors.

In addition to these positive regulators, there are negative regulators of secondary cell wall development and lignin biosynthesis. These include MYB transcription factors belonging to subgroup 4 (MYB3, MYB4, MYB7, and MYB32) that act as negative regulators in lignin biosynthesis (Jin et al., 2000; Preston et al., 2004; Wang and Dixon, 2012; Zhou et al., 2017). In cotton, *GhMYB4* down-regulates lignin biosynthetic genes, including *C4H*, *4CL*, and *CAD* (Xiao et al., 2021). Similarly, poplar *PtoMYB156* and banana *MusaMYb31* are involved in negatively regulating lignin biosynthesis (Tak et al., 2017; Yang et al., 2017). The negative regulation of lignin biosynthetic genes by MYB transcription factors can be explained by the EAR domain in their C-termini. NIGHT LIGHT-INDUCIBLE AND CLOCK-REGULATED 1 (LNK1) and LNK2 were found to be corepressors that physically interact with the EAR domain of MYB3 to enhance the repressive activity of MYB3 on *C4H* expression (Zhou et al., 2017). MYB4, MYB7, and MYB32 also suppress expression of their upstream regulator, *SND1* (Wang et al., 2011). These mechanisms constitute a negative feedback regulatory loop.

**Figure 2:**
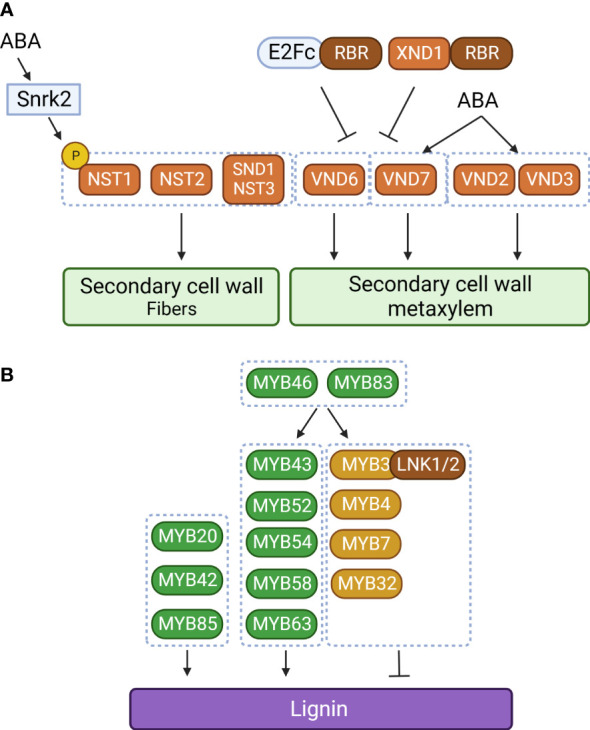
The NAC–MYC transcriptional network of lignin biosynthetic genes in plants. **(A)** Regulation of secondary cell wall development in xylem cells and fibers. ABA regulates xylem cell and fiber development through phosphorylation of NAC SECONDARY WALL THICKENING PROMOTING FACTOR1 (NST1) and transcriptional activation of VASCULAR-RELATED NAC DOMAIN 2 (VND2), VND3, and VDN7. SnrK2, SNF1-related protein kinase 2; RBR, RETINOBLASTOMA-RELATED; XND1, XYLEM NAC DOMAIN 1. **(B)** Transcriptional regulation of lignin biosynthesis. LNK1, NIGHT LIGHT-INDUCIBLE AND CLOCK-REGULATED 1; Orange box, NAC transcription factor; green and light brown boxes, MYB transcription factor; dark brown box, transcriptional corepressor; black arrow, activation; black line with bar, repression. The figure was created with Biorender (http://biorender.com).

In addition to the NAC–MYB regulatory network, other groups of transcription factors are also involved in lignin biosynthesis. OsIDD2, a C2H2 zinc finger transcription factor, acts as a repressor for lignin deposition by repressing expression of *OsCAD2* and *OsCAD3* (Huang et al., 2018). In Arabidopsis, E2Fc was proposed as an upstream regulator of *VND7* (Taylor-Teeples et al., 2015). Since E2Fc forms a repressor complex with the RETINOBLASTOMA-RELATED (RBR) protein, the E2Fc–RBR complex inhibits *VND6* and *VND7* expression, but a moderate E2Fc level activates *VND7* expression (Taylor-Teeples et al., 2015). Thus, E2Fc can act as both an activator and a repressor to regulate *VND7* gene expression (Taylor-Teeples et al., 2015). In addition to VND6 and VND7, E2Fc also binds to the promoters of phenylpropanoid and lignin formation genes (*C4H*, *CCoAOMT*, *CAD4*, and *LAC4*). Interestingly, it has also been reported that the NAC-type transcription factor XYLEM NAC DOMAIN1 (XND1) interacts with RBR and acts as a repressor of xylem development (Zhao et al., 2017). The XND1–RBR repressor complex negatively regulates expression of *NST1, NST2, VND6*, and *VND7* (Zhao et al., 2017). In addition, XND1 represses VND6-mediated transcriptional regulation by sequestering VND6 in the cytoplasm (Zhong et al., 2021). These findings suggest that the transcription factor–RBR complex constitutes an additional regulatory network for secondary cell wall formation and lignin deposition (**Figure 2**).

Through targeting MYB transcription factors, certain microRNAs are also involved in the post-transcriptional regulation of lignin biosynthesis. For one, miR828 negatively regulates lignin biosynthesis by targeting *MYB171* and *MYB011*, which are activators of *PAL1* and *CCR2* expression (Wang et al., 2022). Overexpression of *miR858* down-regulates expression of its target genes (*MYB11*, *MYB12*, and *MYB111*), resulting in up-regulation of lignin biosynthetic genes and down-regulation of flavonoid biosynthetic genes (Sharma et al., 2016). Because the two pathways share a precursor, the miR858–MYB module can change the metabolic flow between flavonoid and phenylpropanoid biosynthetic pathways. These results suggest that miR858 promotes lignin biosynthesis, whereas miR828 inhibits it, by targeting different sets of MYB transcription factors involved in lignin biosynthesis. Recently, poplar miR395c was found to be involved in both sulfate metabolism and secondary cell wall development (Liu et al., 2022). Overexpression of xylem-specific *miR395c* increased secondary xylem width but decreased secondary cell thickness. It was also found that *miR395c* overexpression down-regulated *MYB46* expression (Liu et al., 2022), thereby inhibiting expression of lignin biosynthetic genes. LAC activity is also post-transcriptionally regulated by miRNAs. *miR397* has been found to target *LAC4* to reduce lignin deposition in Arabidopsis, poplar, and cotton plants (Lu et al., 2013; Wang et al., 2014; Wei et al., 2021). Similarly, maize miR528 negatively regulates lignin deposition in vascular tissues by targeting *ZmLAC3* and *ZmLAC5* (Sun et al., 2018). These instances of miRNA-mediated regulation of the lignin biosynthetic pathway describe a fine-tuning of lignin biosynthesis in response to developmental cues and environmental stresses in plants.

## Importance of lignin in the drought responses of plants

Lignin deposition in vascular cells is important not only for long-distance water transport but also for recovery from drought. In plants, the concentration of lignin is higher in vascular tissue such as the secondary cell wall of TEs as well as primary cell walls and cell corners of xylem cells (Serk et al., 2015). To address the impact of drought on lignin deposition, changes in lignin deposition have been investigated in plants (**Table 1**). Overall, drought generally increased total lignin content in several plant species (Fan et al., 2006; Gu et al., 2020; Sharma et al., 2020; Liu et al., 2021b). Analysis of tissue-specific lignin deposition showed that lignin accumulates in xylem cells or xylary fibers in response to drought treatment (Fan et al., 2006; Srivastava et al., 2015; Sharma et al., 2020; Liu et al., 2021b). It has been proposed that increased lignification in xylem cells enhances drought tolerance in plants. In normal conditions, water is driven from the soil, through the plant, and to the atmosphere through differences in water potential. Drought decreases the water potential of soil, creating a deficit between water supply in the roots and demand from the leaves. Increases in this difference in water potential cause the inward collapse of TEs, disrupting plant hydraulic conductivity (Cochard et al., 2004; Voelker et al., 2011). The accumulation of lignin increases the strength of TEs, resulting in enhanced resistance to drought-mediated xylem collapse. Recently, Menard et al. reported that the degree of lignin deposition controls the biomechanics of TEs, which are important for maintaining water transport systems during and after drought. The authors found that TEs can withstand drought better than parenchymatic cells. Moreover, the degree of post-mortem lignification of TEs was positively correlated with TE resistance to collapse induced by air drying (Ménard et al., 2022). In addition, the composition of lignin polymers is another potentially important determinant of drought tolerance (Pesquet et al., 2019). In particular, G_CHO_ in TE cell walls was found to significantly affect plant recovery from drought. Higher accumulation of G_CHO_ in a *cad4 cad5* double mutant increased drought tolerance by reducing inward collapse and enhancing recovery of the initial structure of TEs (Ménard et al., 2022). These findings suggest that modulating the ratio of lignin subunits, as well as lignin content, is important for drought tolerance in plants. Further detailed analyses investigating the impact of drought on lignin chemistry are required to expand our understanding of physical adaptation to drought in plants.

**Table 1:** Effect of drought treatments on lignin deposition in plants.

Plant species	Treatment	Lignin content	Lignified tissue	Genes involved in lignin deposition	Reference
**Tea tree** **(*Camellia sinensis*)**	Withholding water	Increased	N.D.	*CsPAL, CsCCR*, *CsPRX*	Gu et al., 2020
**Chickpea** ***(Cicer arietinum)***	Withholding water	Increased	Roots (metaxylem, protoxylem)	*CaLACs*	Sharma et al., 2020
**Oriental melon** **(*Cucumis melo*)**	8% PEG6000	Increased	Stems (metaxylem, protoxylem)	*CmPAL,CmC4H, Cm4CL, CmCCR, CmCOMT, CmPOD, CmLAC*	Liu et al., 2021b
**Maize** **(*Zea mays*)**	PEG6000 (−0.5 MPa)	Increased	Roots (xylem fiber)	*ZmCCR*	Fan et al., 2006
**Maize** **(*Zea mays*)**	16% PEG6000	Higher in drought tolerant inbred lines	N.D.	*ZmCAD*, *ZmCOMT*	Hu et al., 2009
**White popinac (*Leucaena leucocephala*)**	1% mannitol	Higher than control plants	Stems and roots (xylem and xylem fibers)	*LlCCR*	Srivastava et al., 2015
**Arabidopsis** **(*Arabidopsis thaliana*)**	1 µM ABA	Increased	Roots (inner metaxylem position)	*VND2, VND3, VDN7*	Ramachandran et al., 2021

N.D., Not determined.

## Drought-mediated regulation of lignin biosynthesis

In plants, drought triggers a series of physiological and biochemical changes, some of which alter lignin biosynthesis and deposition. When placed under drought or water deficit conditions, several plants have shown an increase in lignin content that is potentially achieved through transcriptional regulation of phenylpropanoid or lignin formation genes (Gu et al., 2020; Sharma et al., 2020; Liu et al., 2021b) (**Table 1**). For example, Arabidopsis *CCoAOMT* is induced under drought conditions, and its mutation caused plants to become hypersensitive to drought (Chun et al., 2021). In rice, drought induced the up-regulation of *OsCCR10*, which can mediate production of G subunits *via* reduction of feruloyl-CoA (Bang et al., 2022). In watermelon, *CmCAD2* and *CmCAD3*, which are responsible for the biosynthesis of S and G subunits, are important for drought tolerance (Liu et al., 2020). In maize, drought enhanced lignin deposition in roots by inducing *CCR1* and *CCR2* expression (Fan et al., 2006). Drought stress also induced expression of phenylpropanoid biosynthesis genes such as *CAD* and *CYP* and triggered the accumulation of associated proteins in maize leaves (Hu et al., 2009). Similarly, drought treatment enhanced CCR protein accumulation and stem lignin deposition in *Leucaena leucocephala* (Srivastava et al., 2015). Likewise, drought acts as an input for the activation of several phenylpropanoid biosynthesis genes. However, relatively little is known about the molecular signaling involved in drought-mediated lignin deposition in plants.

It has been proposed that ABA acts as a link between drought and secondary cell wall development (Liu et al., 2021a; Ramachandran et al., 2021). ABA treatment is known to enhance xylem differentiation by inducing expression of NAC transcription factors related to secondary cell wall formation (Jensen et al., 2010; Chen et al., 2020; Ramachandran et al., 2021). Ramachandran et al. (2021) found that *VND2*, *VND3*, and *VND7* are involved in ABA-mediated changes to xylem development. ABA-dependent early inner xylem cell differentiation was not visible in *vnd2 vnd3* and *vdn1 vnd2 vdn3* mutant plants, suggesting that *VND2* and *VND3* are required to regulate the plasticity of the xylem differentiation rate under drought conditions (Ramachandran et al., 2021). On the other hand, *VND7* mediates the ABA-dependent conversion of xylem cells into a protoxylem morphology. Enhanced xylem differentiation may reduce the chance that water transport is interrupted under drought conditions (Tang et al., 2018). In addition to affecting xylem differentiation genes, exogenous ABA treatment also induced the expression of genes related to cellulose [*CELLULOSE SYNTHASEA4* (*CESA4*), *CESA7*, and *CESA8*] and lignin formation (*LAC11* and *LAC17*), resulting in ectopic lignin deposition in cotyledons and stimulation of the development of lignified xylem cells in roots (Ramachandran et al., 2021). On the other hand, inhibition of ABA synthesis or disruption of ABA signaling reduced expression of multiple phenylpropanoid biosynthetic genes (*PAL1*, *C4H*, and *CCR1*) and their upstream regulators (*SND1*, *NST1*, *MYB46*, and *MYB83*), resulting in reduced lignin deposition and secondary cell wall development (Liu et al., 2021a). These observations propose a possible connection between ABA signaling and lignin deposition in plants. Specifically, ABA-dependent phosphorylation seems to play a central role in ABA-mediated lignin deposition. ABA-dependent Snrk2 kinases (Snrk2.2, Snrk2.3, and Snrk2.6) physically interact with NST1, a master regulator of secondary cell wall development and lignin deposition. In this interaction, ABA-dependent Snrk2 kinases phosphorylate NST1 at Ser316, leading to the activation of NST1 (Liu et al., 2021a). The Ser residues are highly conserved among NST1 in dicots but not in monocots, suggesting that ABA–Snrk2-dependent regulation of xylem differentiation and corresponding lignin deposition might function exclusively in dicots.

In addition to playing a role in vascular tissue development and lignin deposition, ABA is also involved in signaling related to Casparian strips (Wang et al., 2019). The deposition of the Casparian strip in the endodermal cell wall prevents apoplastic diffusion of water and solutes (Geldner, 2013). Lignin deposition in Casparian strip is controlled by Casparian strip membrane domain proteins (CASPs), peroxidase, and ENHANCED SUBERIN1 (ESB1) (Roppolo et al., 2011; Hosmani et al., 2013; Lee et al., 2013). To maintain Casparian strip functionality, plants operate Schengen pathway, which consists of CASPARIAN STRIP INTEGRITY FACTORS 1 (CIF1), CIF2, and SCHENGEN3 (SGN3), to boost Casparian strip deposition (Doblas et al., 2017; Nakayama et al., 2017; Fujita et al., 2020). Once the Casparian strip is sealed, CIF signal peptides are blocked, thus inactivating the Schengen pathway. When a Casparian strip is damaged, internal CIFs are released, activating the Schengen pathway. Mutation of *MYB36* or *ESB1* was reported to produce impaired Casparian strip, reflecting that the Schengen pathway was constitutively active, while mutation of *SGN3* resulted in discontinuous lignification of Casparian strip (Reyt et al., 2021). In an *myb36* mutant, lignification was observed at the cell corners of endodermal cells. However, the cell corner–specific lignification was no more visible in *sgn3 myb36* double mutants. These observations indicate that *SNG3* mediates compensatory lignification in cell corners and that *MYB36* mediates endodermal lignification (Reyt et al., 2021). Further, the lack of lignification in the Casparian strip or cell corners significantly increased the permeability of root apoplasts, indicating that lignification in Casparian strip is crucial for establishing a diffusion barrier. Interestingly, a *sgn3 myb36* double mutant displayed severe growth retardation under low-humidity conditions, but grew normally under high humidity, suggesting that reduced leaf transpiration is a key mechanism in mitigating the loss of Casparian strip integrity by maintaining root ionic homeostasis (Reyt et al., 2021). Since leaf transpiration is largely controlled by ABA, it is possible that ABA links loss of Casparian strip integrity and stomatal closure. Wang et al. (2019) found that ABA is involved in the regulation of stomatal closure mediated by the Schengen pathway. Enhanced stomatal closure in an *esb1-1* mutant was suppressed by introduction of a dominant negative allele of the ABA signaling regulator ABA-INSENSITIVE 1 (ABI1) (Wang et al., 2019). These observations suggest that ABA is required not only for xylem cell differentiation and lignin deposition but also for the regulation of stomatal closure induced by the loss of diffusion barrier Casparian strip.

## Utilizing the lignin biosynthesis transcriptional network to enhance drought tolerance

The effects of drought on lignin deposition have been extensively studied at the level of transcriptional regulation of lignin biosynthetic genes. These studies have reported that expression of lignin biosynthetic genes and their upstream regulators is induced by drought and ABA, and that their overexpression confers drought tolerance by increasing lignin deposition in plants (Lee et al., 2016; Tu et al., 2020; Bang et al., 2022; Jung et al., 2022) (**Table 2**). For example, the increased expression of some lignin biosynthetic genes (*PAL*, *4CL*, *CCoAOMT*, and *CAD*) has been observed in wild watermelon (*Citrullus lanatus*) during the intermediate and final stages of drought stress (Yoshimura et al., 2008; Moura et al., 2010). Overexpression of *CmCAD2* or *CmCAD3* in oriental melon enhanced drought tolerance by stimulating lignin accumulation (Liu et al., 2020). *CmCAD2* and *CmCAD3* are also required for formation of the Casparian strip and sclerenchyma cells in roots, suggesting that overexpression of *CmCAD2* or *CmCAD3* contributes to drought tolerance *via* stabilizing internal water content (Liu et al., 2020). Similarly, overexpression of rice *OsCCR10* conferred drought tolerance through enhanced lignin deposition, whereas CRISPR/Cas9-mediated mutation of *OsCCR10* decreased lignin accumulation and drought tolerance (Bang et al., 2022). *OsCCR10* is predominantly expressed in roots and further up-regulated by drought and ABA. An *in vitro* enzyme assay showed that OsCCR10 generates H and G subunits by reducing coumaryl-CoA and feruloyl-CoA. Consistent with the *in vitro* analysis, *OsCCR10*-overexpressing plants accumulated more H and G units. Interestingly, ectopic expression of *OsCCR10* increased lignin deposition in roots, but not in leaves and stems. This result can be explained by the reduced substrate availability in leaves and stems due to tissue-specific expression of other lignin biosynthetic genes (Barros et al., 2015). Drought-inducible expression of *CCR* genes is mediated by NAC- and CCCH-type transcription factors. In rice, overexpression of the drought-inducible *OsNAC5* gene greatly increased root-mediated drought tolerance by enlarging xylem and aerenchyma (Jeong et al., 2013). *OsCCR10* has been reported to act as a direct target of OsNAC5, suggesting that OsNAC5-mediated up-regulation of *OsCCR10* contributes to increased xylem development through the accumulation of lignin (Bang et al., 2022). It has also been reported that drought treatment promoted the accumulation of lignin in *Populous ussuriensis* (Li et al., 2022). They proposed that *PuC3H35*, which encodes a CCCH-type transcription factor and whose expression was induced in roots under drought conditions, positively regulates lignin accumulation in *P. ussuriensis.* Specifically, PuC3H35 stimulated lignin accumulation through direct activation of *early Arabidopsis Aluminum induced 1* (*PuEARLI1*) expression. Like Arabidopsis *EARLI1* (Shi et al., 2011), *PuEARLI1* acts as a positive regulator of *PuCCR* genes (*PuCCR1*, *PuCCR2*, *PuCCR16*, and *PuCCR17*). Overexpression of either *PuC3H35* or *PuEARL1* conferred drought tolerance to plants by increasing lignin accumulation in roots (Li et al., 2022).

**Table 2:** Modification of lignin deposition and its effect on drought tolerance.

Plant species	Gene	Lignin content	Drought tolerance	Lignified tissue	Genes involved in lignin deposition	Reference
**Rice** **(*Oryza sativa*)**	*OsERF71* (overexpression)	Increased	Increased	Roots (between metaxylem and stele)	*OsPAL*, *OsC4H*, *OsCCR1, OsCCR10*, *OsCAD*, *OsPRX*	Lee et al., 2016
**Rice** **(*Oryza sativa*)**	*OsNAC17* (overexpression)	Increased	Increased	Leaves and roots	*OsPAC7, OsCCR29, OsPRX22*, *OsPRX131*, *OsCAD8D*	Jung et al., 2022
**Rice** **(*Oryza sativa*)**	*OsCCR10* (overexpression)	Increased	Increased	Stems and roots (metaxylem, xylem fiber, sclerenchyma)	*OsCCR10*	Bang et al., 2022
**Rice** **(*Oryza sativa*)**	*OsTF1L* (overexpression)	Increased	Increased	Shoots (epidermis, sclerenchyma, xylem)	*OsPRX2*, *OsPRX22*, *OsPRX38*, *OsCAD6*, *OsCAD7*, *OsCOMTL5*	Bang et al., 2019
**Grapevine** **(***Vitis vinifera)*	*VlbZIP30* (overexpression)	Increased	Increased	Stems (secondary xylem)	*VvPRX4, VvPRX72*	Tu et al., 2020
**Oriental melon** **(*Cucumis melo*)**	*CmCAD2* (suppression)	Reduced	Reduced	Stems (xylem)	N.D.	Liu et al., 2020
**Oriental melon** **(*Cucumis melo*)**	*CmCAD3* (suppression)	Reduced	Reduced	Stems (xylem)	N.D.	Liu et al., 2020
**Apple** **(*Malus × domestica*)**	*MdMYB88* (overexpression)	Increased	Increased	Roots	*MdVND6*, *MdMYB46*	Geng et al., 2018
**Apple** **(*Malus × domestica*)**	*MdMYB124* (overexpression)	Increased	Increased	Roots	*MdVND6*, *MdMYB46*	Geng et al., 2018
***Populus ussuriensis***	*PuC3H35* (overexpression)	Increased	Increased	Roots (xylem)	*PuEARL11*	Li et al., 2022
***Foxtail millet*** **(*Setaria italica*)**	*SiMYB56* (overexpression)	Increased	Increased	N.D.	*OsPAL, OsC4H, Os4CL5, OsCCR10, OsCAD, OsF5H1*	Xu et al., 2020
***Cassava* (*Manihot esculenta*)**	MeRAV5 (suppression)	Reduced	Reduced	N.D.	*MeCAD*	Yan et al., 2021
**Arabidopsis** **(*Arabidopsis thaliana*)**	*AtCAD4, AtCAD5*, *AtFAH1* (knockout)	Reduced (increased X_CHO_)	Increased	N.D.	*AtCAD4, AtCAD5*, *AtFAH1*	Ménard et al., 2022

N.D., Not determined.

In addition to up-regulating individual lignin biosynthetic genes, overexpressing certain transcription factors that induce lignin accumulation has successfully enhanced drought tolerance in several plant species. For example, in rice, overexpression of the drought-induced *OsERF71* enhanced lignin deposition and up-regulated several genes involved in phenylpropanoid biosynthesis and lignin formation, such as *PAL*, *C4H*, *CCR*, *CAD*, and *PRX* (Lee et al., 2016). Among these genes, *CCR*, *C4H*, and *CAD* were found to be direct targets of OsERF71 (Lee et al., 2016). In apple, *MdMYB88* and *MdMYB124* were identified as positive regulators of root architecture and hydraulic conductivity under long-term drought stress. Overexpression of *MdMYB88* or *MdMYB124* enhanced drought tolerance by promoting lignin deposition and xylem cell formation (Geng et al., 2018). Changes to lignin deposition and xylem development are mediated by up-regulation of *MdVND6* and *MdMYB46*. *Setaria italica SiMYB56* also promoted drought tolerance by activating expression of the phenylpropanoid genes *4CL5* and *F5H1* (Xu et al., 2020). In addition to these transcription factors regulating lignin biosynthesis, transcription factors involved in lignin formation can also be used to develop drought tolerant plants. In cassava plants, silencing of *MeRAV5* reduced drought tolerance *via* the accumulation of hydrogen peroxide and decrease in lignin (Yan et al., 2021). In this case, MeRAV5 physically interacted with MePOD to increase its enzymatic activity. On the other hand, two drought-induced NAC transcription factors, OsNAC17 and VvNAC17, promoted lignin accumulation by regulating *PRX* genes in rice and grapevine, respectively (Tu et al., 2020; Jung et al., 2022). Similarly, OsTF1L, a rice HD-Zip transcription factor, also directly up-regulated expression of *PRX* genes and conferred drought tolerance when overexpressed (Bang et al., 2019). In addition, VlbZIP30 is known to bind to the G-box in *PRX* promoters to promote lignin formation (Tu et al., 2020). These findings indicate that drought-mediated regulation of the polymerization and biosynthesis of lignin can be used to improve drought tolerance in plants.

## Concluding remarks and future perspectives

As a result of biochemical and genetic studies, the lignin biosynthetic pathway has been well established. Now, research efforts have turned toward elucidating the molecular regulatory mechanisms of lignin biosynthesis and deposition to better understand the physiological changes caused by developmental cues and environmental stresses. The lignin biosynthesis pathway is complex and controlled by various internal and external signals. Currently, the NAC–MYB transcriptional regulatory network is understood to be a master switch in secondary cell wall development and lignin biosynthesis. At the same time, it is obvious that there exist additional regulatory branches that modulate the NAC–MYB master switch in response to developmental cues. However, how these external signals enter into the lignin biosynthetic pathway is poorly understood. The discovery that ABA signaling regulates the NST1-mediated transcriptional network *via* phosphorylation represents a huge step toward linking lignin biosynthesis with abiotic stresses (Liu et al., 2021a). It is worth noting that SnRK-mediated phosphorylation of NST1 seems to be conserved only in dicot plants. This trend raises the question of how monocots regulate lignin biosynthesis in response to abiotic stress. In addition to phosphorylation, S-nitrosylation and ubiquitination can participate in regulating the lignin biosynthesis transcriptional network (Kawabe et al., 2018; Zheng et al., 2019; Sulis and Wang, 2020). Further identification of posttranslational regulation associated with lignin biosynthesis will improve our understanding of developmental and environmental regulation of this pathway.

Even though increased lignin deposition is often beneficial for plant survival (Lee et al., 2019), changes in lignin content can also hinder plant growth and development (Bonawitz and Chapple, 2013; Perkins et al., 2020). For example, it has been reported that *MYB58*- and *MYB63*-overexpressing plants showed growth arrest due to overaccumulation of monolignol glycosides. However, this growth retardation was rescued by ectopic expression of *LAC4* or *LAC17* in Arabidopsis (Perkins et al., 2020). These results suggest that growth limitations brought about by modulation of the lignin biosynthetic pathway can be mitigated through co-activation of rate-limiting enzymes. Therefore, genetic engineering that enhances lignin deposition without inducing growth retardation and yield penalties can be accomplished by systemic modulation of lignin biosynthesis and polymerization pathways. However, there also exist several successful cases, especially in rice, in which overexpression of lignin biosynthetic enzymes or their transcriptional regulators increased drought tolerance without causing growth retardation and yield penalties (Lee et al., 2016; Bang et al., 2022). These cases suggest that the capacity for monolignol biosynthesis and polymerization may differ between plant species. We must also consider that lignin biosynthesis is tightly connected with the biosynthesis of other secondary metabolites (Vanholme et al., 2012; Bonawitz et al., 2014; Lam et al., 2022). When metabolic flow is driven into lignin biosynthesis, it can decrease the content of flavonoids or stilbenes, which are important for plant growth and environmental adaption. For example, suppression of *CmCAD2* and *CmCAD3* resulted in increase of cinnamic acid and ferulic acid, which are important precursors for phenylpropanoid derivatives (Liu et al., 2020). In addition, since *cis*-cinnamic acid acts as an inhibitor for auxin efflux, the accumulation of *cis*-cinnamic acid in plants alters auxin accumulation (Steenackers et al., 2017). Endogenous or exogenous auxin increases drought tolerance of plants by regulating root architecture, ABA-responsive gene expression, and reactive oxygen species metabolism (Shi et al., 2014; Sadok and Schoppach, 2019). These results suggest that metabolic consequences should be considered when modulating lignin deposition through the phenylpropanoid biosynthesis pathway. Metabolic profiling of lignin products and other related secondary metabolites, together with growth and yield analyses, will strengthen the value of lignin-mediated drought tolerant crop development.

## Author contributions

SJC and JSS planned and designed all aspects of the manuscript. SJC, ZL, EJ, SK, and JSS collected information and wrote the manuscript. All authors contributed to the article and approved the submitted version.
